# Silver Nanoparticles for Enhancing the Efficiency of Micropropagation of Banana (*Musa acuminata* L.)

**DOI:** 10.21315/tlsr2023.34.2.8

**Published:** 2023-07-21

**Authors:** Samih M. Tamimi, Halima Othman

**Affiliations:** Department of Biological Sciences, The University of Jordan, Queen Rania St, Amman, Jordan

**Keywords:** Banana, *Musa acuminata*, Silver Nanoparticles, Micropropagation

## Abstract

Silver nanoparticles (AgNPs) have numerous applications in plant biotechnology. The unique biological activities of AgNPs in reducing microbial contamination and promoting *in vitro* plant growth have encouraged their use in the development of novel culture systems for the *in vitro* cultivation of several plant species. In this study, the influence of (80 nm–100 nm) AgNPs on the micropropagation of banana was examined by incorporating AgNPs into shoot multiplication and rooting media at concentrations of 3 mg/L–15 mg/L. Biometric parameters for shoot multiplication (number of shoots/explant, shoot length and leaf surface area) and root development (number of roots/explant and root length) were analysed. In addition, shoot chlorophyll content, proline content and the possible impact of lipid peroxidation on membrane stability of plantlets were estimated. The results showed that all concentrations of AgNPs stimulated shoot growth and enhanced root development. The highest response was observed in media supplemented with 12 mg/L AgNPs. This optimal level of AgNPs caused a threefold increase in shoot growth parameter and a similar increase in root numbers/shoot and root length. Treatment with AgNPs at 12 mg/L also increased chlorophyll and proline content of shoots by 25% and 120% over control, respectively. Although the application of AgNPs increased the level of lipid peroxidation in shoots, it however, had a limited influence on membrane stability index. These results suggested that the administration of AgNPs to culture media can be effectively utilised for the enhancement of banana micropropagation with minimal toxic effects.

**Highlights**

- The effects of (80 nm–100 nm) silver nanoparticles (AgNPs) applied toculture media on the growth responses of micropropagated banana (*Musaacuminata* L.) cultivar (Grand Nain) were investigated.
- The administration of AgNPs to banana culture media can be effectively utilised for the enhancement of banana micropropagation.
- AgNPs as a component of culture media had low toxicity on micropropagated banana plants.

## INTRODUCTION

The technique of *in vitro* micropropagation is one of the most promising options for providing large number of virus free and genetically uniform banana planting material. Although the micropropagation of banana is well established and several successful *in vitro* propagation protocols have been published (for a recent review see Eustache *et al*. 2021), continuous efforts are being made to increase the efficiency of the system through improvement in culture media and techniques. Some of the problems which need to be addressed in order to improve the micropropagation of banana include low number of shoots produced per explants and the short shoots obtained during multiplication. Apparently, an increase in shoot multiplication rate is desirable and requires the search for more effective protocols for the micropropagation of this plant.

With the advancement of nanotechnology, nanomaterials are beginning to be employed in different areas of science, including plant tissue culture. Among nanomaterials, silver nanoparticles (AgNPs) have been demonstrated to improve the *in vitro* multiplication of Banana (El-Mahdy *et al*. 2019; Huong *et al*. 2021). In fact, AgNPs is considered by many as a biostimulator (Ruffini Castiglione & Cremonini 2009) emphasising its promising role in the improvement of *in vitro* propagation of plants.

However, the use of nanoparticles in tissue culture is still a new approach, which needs further study and research for proper understanding and implementation. For instance, it is reported that ionic silver released from AgNPs may induce phytotoxicity to cultured plants such as cell membrane damage and generation of reactive oxygen species (ROS) (Qian *et al*. 2013; McShan *et al*. 2014). In addition, some studies suggested that the phytotoxicity of AgNPs is size and concentration dependent and that smaller AgNPs are more toxic to plants (Geisler-Lee *et al*. 2012; Gorka *et al*. 2015; Kong *et al*. 2020). Unfortunately, all reported studies on the influence of AgNPS on the micropropagation of banana was carried out using small sized AgNPs (25 nm–30 nm) (Huong *et al*. 2021; El-Mahdy *et al*. 2019). To the best of our knowledge, no previous work was conducted on the influence of larger AgNPs on the micropropagation of banana. The aim of this study was therefore, to examine the *in vitro* shoot multiplication and root growth responses of banana (*Musa acuminata* L.) cultivar (Grand Nain) to different concentrations of (80 nm–100 nm) AgNPs applied to culture media. Furthermore, the possible influence of the AgNPs supplemented to culture media on chlorophyll content, proline production, lipid peroxidation and membrane integrity of the *in vitro* cultured banana plants were also investigated.

## MATERIALS AND METHODS

### Plant Material, Culture Medium and Conditions

Shoot tip explants of banana (*Musa acuminata* L.) (cultivar Grand Nain) were excised from young suckers grown in pots. Explants were surface sterilised with 75% ethanol for 50 s followed by 30 min with 40% commercial bleach (Clorox 5.75% NaOCl) to which few drops of Tween-20 were added. After complete washing with sterile distilled water, explants were trimmed to final size of 10 mm to 15 mm in the laminar flow cabinet. For culture initiation, explants were cultured in screw-capped glass vessels containing 30 mL of initiation media composed of MS basal salts (Murashige & Skoog 1962) supplemented with sucrose (40 g/L), thiamine (0.1 g/L), benzylaminopurine (BAP) (12 μM), indole-3-acetic acid (IAA) (3 μM) and cystein HCl (40 mg/L). Medium was solidified with 2 g/L gelrite (Sigma Chemical Co., St. Louis) and its pH was adjusted to 5.8 before autoclaving at 121°C for 15 min. All cultures were incubated at 25°C under 16 h photoperiod for 4 weeks. Light intensity was 35 μmol s^−1^m^−2^.

### Scanning Electron Microscopy (SEM)

The AgNPs used in this study (average particle size 80 nm–100 nm) were obtained from Nanoshel company, Utah, USA. The morphology and size of the nanoparticles were examined under SEM. A small sample of the AgNPS were mounted on aluminium stubs and the mounted specimens were then examined in a scanning electron microscope (VEGA3 TESCAN) at an accelerating voltage of 10 KV.

### Effect of AgNPs on Shoot Multiplication and Growth

To evaluate the influence of AgNPs on shoot multiplication and growth, banana shoot tip explants from the *in vitro* initiated cultures were transferred to multiplication media. Multiplication medium contained MS basal salts, sucrose (40 g/L), thiamine (0.1 g/L), BAP (20 μM) and cystein HCl (40 mg/L) supplemented with different concentrations (0 to 15 mg/L) of AgNPs. Cultures were arranged in a randomised block design with 10 replicates per treatments (three explants per culture bottle) and incubated at 25°C under 16 h photoperiod for 4 weeks. Light intensity was 35 μmol s^−1^m^−2^. After 4 weeks of culture, the number of shoots formed per explants, shoots length (cm) and leaf surface area (cm^2^) were determined.

### Effect of AgNPS on Rooting

For evaluating the effect of AgNPs on *in vitro* rooting, uniform banana shoots formed on multiplication media were excised and transferred to rooting medium. The rooting medium consisted of MS basal slats, sucrose (40 g/L), 2-isopentenyladenine (2iP) (5 μM) and indole-3-butyric acid (IBA) (0.1 μM) supplemented with different concentrations (0 to 15 mg/L) AgNPs. Medium was solidified with 1.8 g/L gelrite and its pH was adjusted to 5.8. Cultures, consisting of 10 replicates per treatment, were incubated at 25°C under 16 h photoperiod. After 3 weeks, the number of roots formed per shoot and root lengths (cm) were estimated.

### Determination of Total Chlorophyll Content

For the determination of leaf chlorophyll content, 0.5 g fresh leaf material of individual treatments was extracted in 5 mL 80% acetone (v/v) for 3 days under dark conditions at 4°C and total chlorophyll content was determined according to Lichtenthaler (1987).

### Determination of Proline Content

Free proline content was determined following the method of Bates *et al*. (1973). Five hundred milligrams of leaf tissue from individual treatments was homogenised in a mortar with pestle using 10 mL of 3% sulfosalicylic acid and subsequently centrifuged at 5,000 g for 10 min. Two milliliter (2 mL) of the supernatant was mixed with 2 mL of acid ninhydrin and 2 mL of glacial acetic acid, shaken well and boiled at 100°C for 1 h. The mixture was cooled on ice and extracted with 5 mL of toluene. The toluene containing the chromophore was separated from the aqueous phase and collected carefully and its absorbance was measured at 520 nm. A standard curve was prepared with analytical grade proline and based on this curve proline content of the samples was calculated.

### Determination of Lipid Peroxidation

Lipid peroxidation was determined by measuring the amount of malondialdehyde (MDA), a product of lipid peroxidation, using the calorimetric method described by Stewart and Bewley (1980). The total of 0.5 g of leaf samples were homogenised in 5 mL of distilled water. An equal volume of 0.5% thiobarbituric acid (TBA) in 20% trichloroacetic acid solution was added and the sample incubated at 95°C for 30 min. The reaction stopped by putting the reaction tubes in the ice bath. The samples then centrifuged at 10,000 ×g for 30 min. The supernatant removed, absorption was read at 532 nm, and the amount of nonspecific absorption at 600 nm was subtracted from this value. The amount of MDA present was calculated from the extinction coefficient of 155 mM^−1^ cm^−1^.

### Effect of AgNPs on Membrane Stability

Electrolyte leakage was used to evaluate membrane stability according to Lutts *et al*. (1996). One gram of fresh leaf tissue from individual treatments were washed with distilled water to remove surface adhered electrolytes and then cut into discs of uniform size. Leaf discs were put in closed test tubes containing 10 mL deionised water, incubated at 25°C for 24 h and subsequently electrical conductivity of the solution (EC1) was recorded. The solution with leaf discs was boiled for 10 min. After cooling and centrifugation, electrical conductivity of the bathing solution was measured (EC2). The electrolyte leakage was calculated as EC1/EC2 and expressed as percentage. Cell membrane stability index (MSI) was calculated as described by Sairam *et al*. (1997) using the following equation:


MSI (%)=[1-(EC1/EC2)]×100

### Statistical Analysis and Presentation of Results

All data were presented as means of all replicates ± standard error. Means were separated by Duncan’s multiple range test (DMRT) (Duncan 1955) at 5% significance level.

## RESULTS

**Figure 1:**
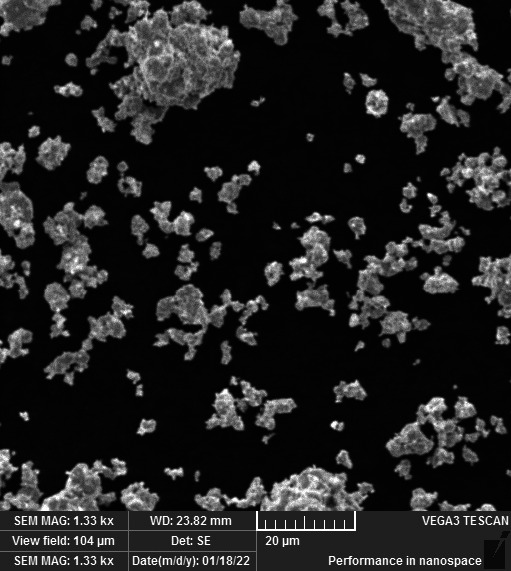
The SEM micrograph of silver nanoparticles.

**Figure 2:**
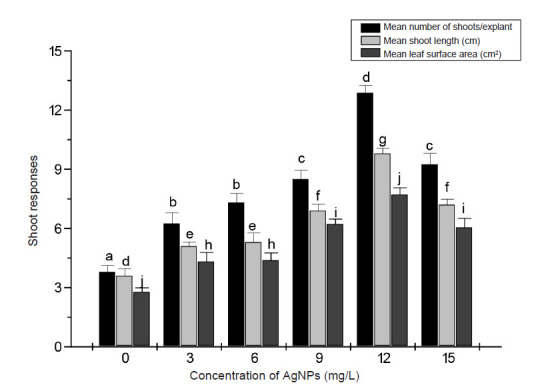
Shoot growth response of banana explants to various concentrations of AgNPs supplemented to shoot multiplication media. Data were collected after 28 days culture and are presented as the means from 10 replicates ± SE. Bars sharing similar letters do not differ significantly (*p* < 0.05).

**Figure 3:**
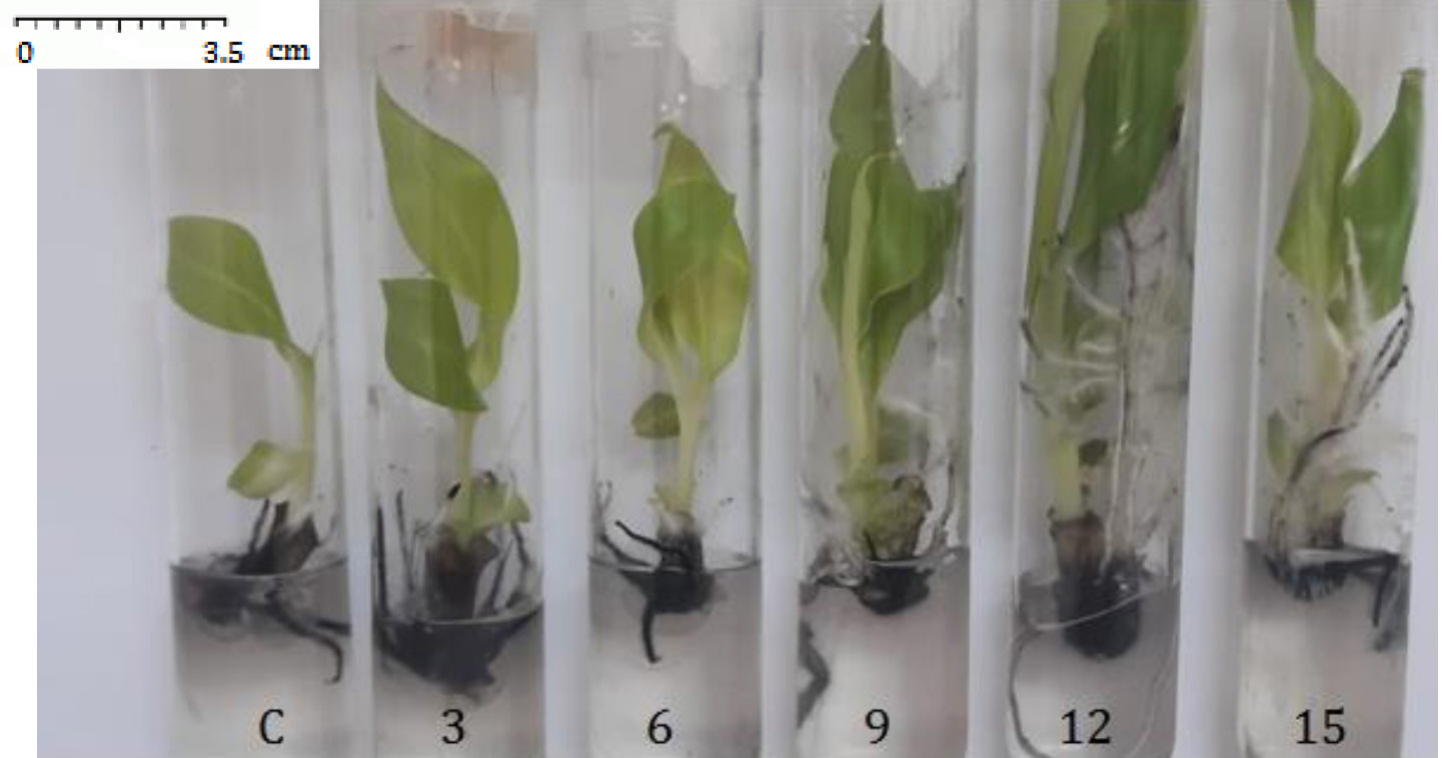
Banana plantlets showing plant growth enhancement when treated with the AgNPs at concentrations of 3 mg/L to 15 mg/L along with control (C).

The image of AgNPs taken by SEM displayed the spherical shape of AgNPs and confirmed that the purchased AgNPs sizes were in the 80 nm–100 nm range (Fig. 1). Exposure of banana shoot explants to different concentrations of the AgNPs resulted in an increase in shoot multiplication proportional to the increase in the concentration of of AgNPs in culture media and reached its highest value in 12 mg/L treatment (Figs. 2 and 3). Multiplication media supplemented with 12 mg/L AgNPs caused approximately threefold increase in growth indicators; number of shoots/explants (12.6), shoots length (9.7 cm) and leaf surface area (7.4 cm^2^) over those of explants cultured in control media [(3.7), (3.4 cm) and (2.6 cm2)], respectively.

**Figure 4:**
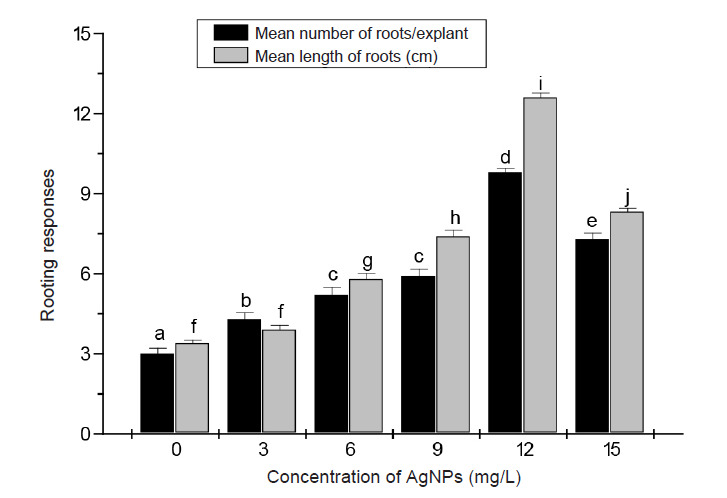
Root growth response of banana explants to various concentrations of AgNPs supplemented to rooting media. Data are collected after 21 days culture and are presented as the means from 10 replicates ± SE. Bars sharing similar letters do not differ significantly (*p* < 0.05).

The rooting response (root number and root length) of the *in vitro* raised banana plants were similarly stimulated by the addition of AgNPs to rooting media (Fig. 4). Shoots cultured on rooting medium supplemented with 12 mg/L AgNPs had the best rooting ability and root growth; number of roots/explant (12.4) and root length (9.5 cm) compared to those cultured on control medium (2.9 cm and 3.2 cm, respectively).

While treatment with AgNPs at 12 mg/L was the optimal for shoot and root growth compared to the remaining treatments, the results of this study showed that further increase in the concentration of AgNPs (15 mg/L) in the cultivation media reduced the observed positive influence on both shoot multiplication and rooting. These findings suggested that concentrations of AgNPs above 12 mg/L might be inhibitory to the *in vitro* growth of banana shoots and roots.

**Figure 5:**
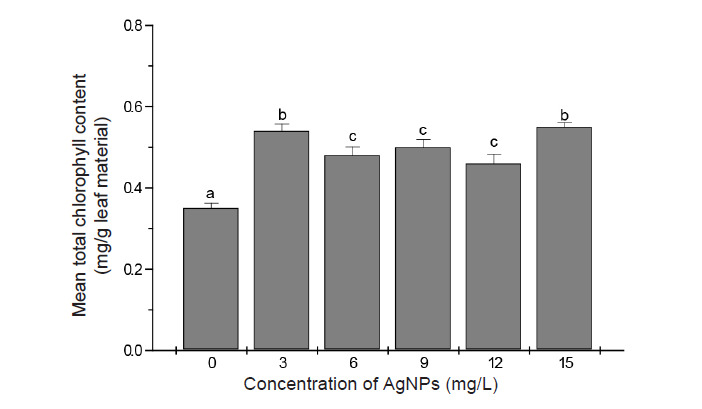
Total chlorophyll content of banana leaves after 28 days culture in shoot multiplication media supplemented with different concentrations of AgNPs. Data are the means from 5 replicates ± SE. Bars sharing similar letters do not differ significantly (*p* < 0.05).

The total leaf chlorophyll content were measured to estimate the possible change in photosynthetic potential of the *in vitro* raised plants as a function of exposure to various levels of AgNPs. In our study, we observed a significant increase in the total chlorophyll content in all the treatment groups in comparison to control treatment (Fig. 5). While AgNPs at 3 mg/L and 15 mg/L resulted in slightly higher increase in chlorophyll content compared to other treatments, the overall trend was an increase in chlorophyll content by approximately 35%–45% over control.

**Figure 6:**
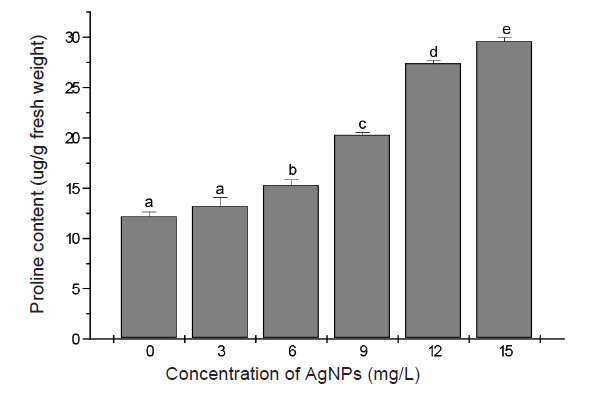
Total proline content of banana leaves after 28 days culture in shoot multiplication media supplemented with different concentrations AgNPs. Data are the means from 5 replicates ± SE. Bars sharing similar letters do not differ significantly (*p* < 0.05).

A gradual increase in the concentration of AgNPs in culture media beyond 3 mg/L resulted in an increase in proline content proportional to the increase in the concentration of AgNPs (Fig. 6). The highest increase in the concentration of proline occurred in plants cultured in media containing 15 mg/L AgNPs. Proline accumulation in response to treatment with AgNPs at 6 mg/L, 9 mg/L and 12 mg/L increased by 22%, 62% and 120% over control, respectively.

**Figure 7:**
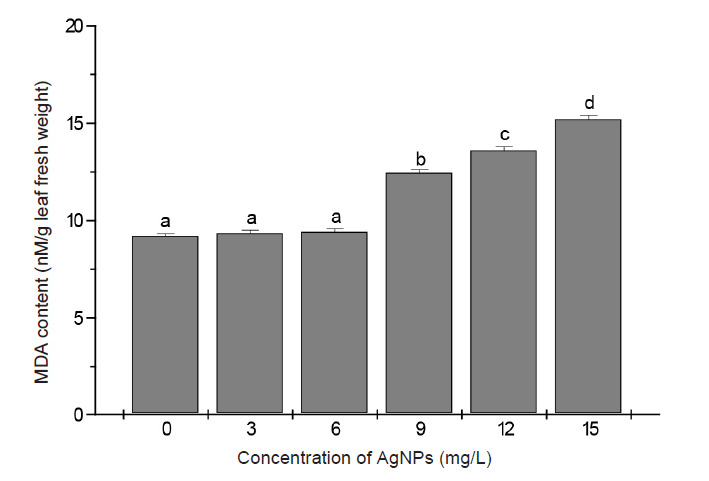
MDA content of banana leaves after 28 days culture in shoot multiplication media supplemented with different concentrations of AgNPs. Data are the means from 5 replicates ± SE. Bars sharing similar letters do not differ significantly (*p* < 0.05).

Lipid peroxidation estimated in terms of MDA content was not altered by treatments with 3 mg/L and 6 mg/L AgNPs compared to control. An increasing trend in MDA content was displayed at higher concentrations of AgNPs. The respective MDA content at the 9 mg/L, 12 mg/L and 15 mg/L AgNPs treatments were 31%, 40.5% and 52% higher than untreated control (Fig. 7).

**Figure 8:**
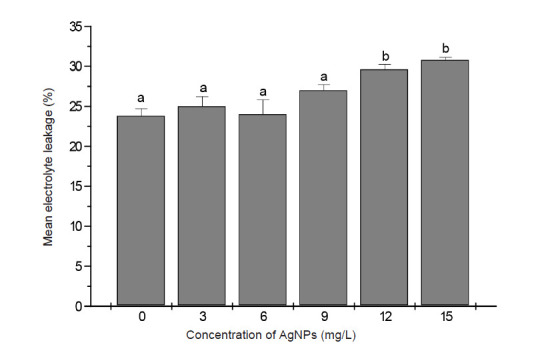
Effect of supplementing shoot multiplication media with different concentrations of AgNPs on electrolyte leakage of banana shoots after 28 days culture. Data are the means from 5 replicates ± SE. Bars sharing similar letters do not differ significantly (*p* < 0.05).

To measure the plasma membrane integrity of AgNPs treated plants, electrolyte leakage analysis was performed and membrane stability indexed (MSI) was determined. According to Fig. 8, no significant difference in electrolyte leakage and MSI in comparison to control plants was detected in leaf tissues of banana plants treated with AgNPs at 3 mg/L and 6 mg/L. Higher concentrations of AgNPs (9 mg/L) increased electrolyte leakage and decreased MSI by 15% relative to control while 12 mg/L and 15 mg/L AgNPs resulted in a higher, but similar increase in electrolyte leakage and reduced MSI by 25% relative to control.

## DISCUSSION

The results of the present study demonstrated that AgNPs in culture medium significantly improved the micropropagation parameters of banana. Administration of doses of AgNPs in the range of 3 mg/L-15 mg/L to shoot multiplication medium stimulated shoot growth and the highest response, (twofold increase) in terms of shoot number/explant, shoot length and leaf surface area was recorded in media supplemented with 12 mg/L. This treatment not only improved shoot multiplication but also stimulated root formation. More roots (threefold higher than control) were produced in explants and a twofold increase in root length were evident in shoots grown in rooting medium supplemented with 12 mg/L AgNPs. These findings suggested that 12 mg/L AgNPs is the optimum for enhancing the micropropagation of banana. Higher doses, however, should be avoided since increasing the concentrations of AgNPs above 12 mg/L restricted shoot and root length and decreased other morphological features. Adverse effects of high concentrations of AgNPs have been previously reported. Zuverza-Mena *et al*. (2016) found that high concentrations of AgNPs reduced shoot and root growth in *Raphanus sativus*. Spinoso-Castillo *et al*. (2017) investigated the development of *Vanilla planifolia* in temporary immersion systems. As in our study, they confirmed toxicity of AgNPs in plants at high concentrations. It is now believed that high doses of AgNPs, block nutrient transportation by ionic channel competition and, reducing shoot and root number and length (Bello-Bello *et al*. 2017).

Despite the difficulty of comparing the results of different studies due to the variety of sizes, shapes and concentrations of AgNPs used, the results of the current study are in agreement with the findings of Huong *et al*. (2021) and El-Mahdy *et al*. (2019) in terms of the positive effect of AgNPs on *in vitro* culture of banana plants. The findings of this study are also in line with previously reported positive effect of small doses and the inhibitory effect of greater concentrations of AgNPs on the *in vitro* growth of potato (Homaee & Ehsanpour 2015), sugarcane (Bello-Bello *et al*. 2017), stevia (Castro-González *et al*. 2019), rose (Ha *et al*. 2020), date palm (El-Kosary *et al*. 2020) and chrysanthemum (Tung *et al*. 2021).

Along with its potential to enhance growth, the results of this study showed that AgNPs significantly increased chlorophyll content of cultured banana shoots. These findings are similar to the observations of Huong *et al*. (2021) who reported an increase in chlorophyll content of banana exposed to AgNPs. An increase in chlorophyll content in response to treatment with small doses of AgNPs was also reported for other plants such as rice (Mirzajani *et al*. 2013), vanilla (Spinoso-Castillo *et al*. 2017) and sugarcane (Bello-Bello *et al*. 2017).

While several suggestions were put forward to provide an explanation for the promotive effect of AgNPs on plant growth and development (Rahmawati *et al*. 2022), one plausible explanation for the positive influence of AgNPs on *in vitro* growth responses of plants are based on its effects on plant hormones. For instance, the property of AgNPs as an ethylene action inhibition makes it widely applicable in plant tissue culture (Aghdaei *et al*. 2012). In addition, the reported increase in the total cytokinin levels of plant tissues exposed to AgNPs (Vinkovic *et al*. 2017) emphasises its role in the growth response to applied AgNPs. Moreover, some studies have demonstrated an increase in the levels of nutrients such as N, Mg and Fe in plants treated with AgNPs (Bello-Bello *et al*. 2017; Spinoso-Castillo *et al*. 2017). It is now believed that the increase in the level of these nutrients is related to the increased chlorophyll synthesis following treatment with AgNPs. Accordingly, this could lead to higher photosynthetic activity and possibly a better plant growth performance.

The administration of AgNPs to plant culture media is generally reported to induce oxidative stress through the generation of ROS (Qian *et al*. 2013; McShan *et al*. 2014). The increase in ROS within membranes can cause considerable damage and disruption of normal cellular activity through destabilisation of cell membranes. MDA content is generally the accepted indicator of membrane lipid peroxidation under ROS action (Su *et al*. 2019). In accordance with previously reported studies confirming the occurrence of lipid peroxidation in different plant species exposed to AgNPs (Bello-Bello *et al*. 2017; Gupta *et al*. 2018) this study showed that increased level of lipid peroxidation (inferred from the high level of MDA content in banana shoots) is associated with increased dose of applied AgNPs. The percentage reduction of membrane stability index (MSI), which is based on the level of electrolyte leakage, is considered to reflect the extent of membrane damage caused by lipid peroxidation. According to Tabaei-Aghdaei *et al*. (2000) a decrease in MSI by more than 50% is taken as an indicator of cell membrane damage. The findings that MSI in banana shoots was reduced only by 15%–25% following the administration of AgNPs to culture media indicated no significant membrane damage of banana shoots by the applied doses of AgNPs.

The remarkable increase of proline content observed in AgNPs treated banana plants is in agreement with previously reported increase of proline levels in plants exposed to AgNPs (Barbasz *et al*. 2016; Shaikhaldein *et al*. 2020). Proline is one of the major compatible solutes produced in plants under stress and is believed to be involved in ROS detoxification (Kumari *et al*. 2017). Therefore, the increase in the accumulation of proline in banana shoots could be a part of a defence mechanisms that maintained membrane stability and protected cellular structures of banana thus, minimising the toxic effects of AgNPs on this plant.

## CONCLUSION

The findings of the present investigation suggested that the addition of 12 mg/L of (80 nm–100 nm) AgNPs as a component of culture media could be a low toxicity efficient strategy for the micropropagation of banana.
